# 1231. *In Vitro* Activity of Aztreonam-Avibactam and Comparator Agents Against Enterobacterales from Patients with Lower Respiratory Tract Infections Collected During the ATLAS Global Surveillance Program, 2017-2019

**DOI:** 10.1093/ofid/ofab466.1423

**Published:** 2021-12-04

**Authors:** Sibylle Lob, Krystyna Kazmierczak, Francis Arhin, Daniel F Sahm

**Affiliations:** 1 IHMA, Inc., Schaumburg, IL; 2 Pfizer Canada, Kirkland, Quebec, Canada

## Abstract

**Background:**

β-lactamase-producing Enterobacterales (Ebact) frequently co-carry resistance to antimicrobials from other classes, limiting treatment options. Avibactam (AVI) inhibits class A, class C, and some class D serine β-lactamases, while aztreonam (ATM) is refractory to hydrolysis by class B metallo-β-lactamases (MBLs). ATM-AVI is being developed for use against drug-resistant isolates of Ebact, especially those co-producing MBLs and serine β-lactamases. This study evaluated the *in vitro* activity of ATM-AVI and comparators against Ebact collected in 2017-2019 from patients with lower respiratory tract infections (LRTI) as part of the Antimicrobial Testing Leadership and Surveillance (ATLAS) program.

**Methods:**

Non-duplicate clinical isolates were collected in 52 countries in Europe, Latin America, Asia/Pacific (excluding mainland China and India), and Middle East/Africa. Susceptibility testing was performed by CLSI broth microdilution and interpreted using CLSI 2021 and FDA (tigecycline) breakpoints. ATM-AVI was tested at a fixed concentration of 4 µg/mL AVI. MDR was defined as resistant (R) to ≥3 of 7 sentinel drugs: amikacin, aztreonam, cefepime, colistin, levofloxacin, meropenem, and piperacillin-tazobactam. PCR and sequencing were used to determine the β-lactamase genes present in all isolates with meropenem MIC >1 µg/mL, and *Escherichia coli*, *Klebsiella* spp. and *Proteus mirabilis* with ATM or ceftazidime MIC >1 µg/mL.

**Results:**

ATM-AVI was active *in vitro* against Ebact isolates from LRTI (MIC_90_, 0.25 µg/mL), with 99.97% of isolates inhibited by ≤8 µg/mL of ATM-AVI, including 100% of isolates that produced MBLs. ATM-AVI tested with MIC_90_ values of 0.5 µg/mL against subsets of cefepime-nonsusceptible (NS), meropenem-NS, amikacin-NS, colistin-resistant, and MBL-positive Ebact (Table). The tested β-lactam comparators showed susceptibility of < 78% against these subsets of resistant isolates.

Results Table

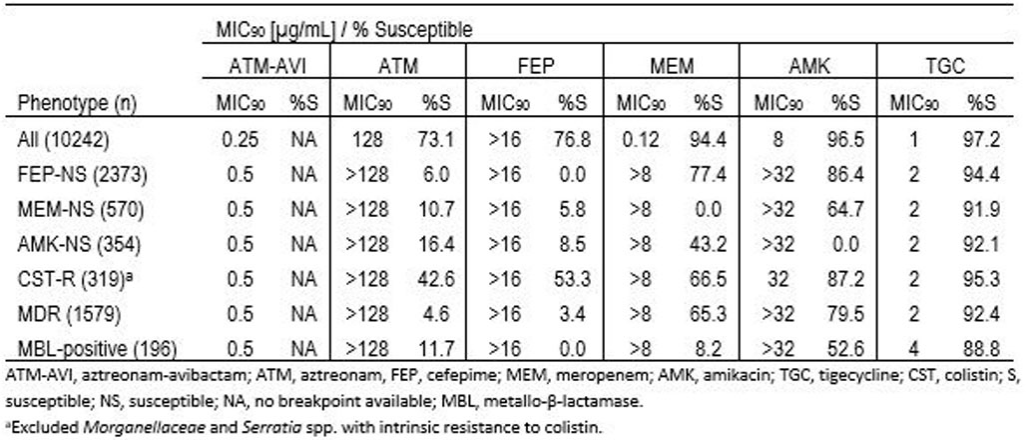

**Conclusion:**

Based on MIC_90_ values, ATM-AVI was the most potent agent tested against drug-resistant and MBL-positive subsets of Ebact collected from LRTI. The promising *in vitro* activity of ATM-AVI warrants further development of this combination for treatment of LRTI caused by drug-resistant Ebact.

**Disclosures:**

**Sibylle Lob, PhD**, **IHMA** (Employee)**Pfizer, Inc.** (Independent Contractor) **Krystyna Kazmierczak, PhD**, **IHMA** (Employee)**Pfizer, Inc.** (Independent Contractor) **Francis Arhin, PhD**, **Pfizer, Inc.** (Employee) **Daniel F. Sahm, PhD**, **IHMA** (Employee)**Pfizer, Inc.** (Independent Contractor)

